# Commentary: Availability of healthy and unhealthy foods in modern retail outlets located in selected districts of Greater Accra Region, Ghana

**DOI:** 10.3389/fpubh.2026.1733568

**Published:** 2026-02-16

**Authors:** Ancy Antony, B. Abirami, Meghna Rajan, T. Vidhya Vijayakumar, Larisha Janet Rodrigues, B. Sreya

**Affiliations:** 1 PSG College of Arts and Science, Coimbatore, India; 2 PSGR Krishnammal College for Women, Coimbatore, India; 3 Joy University, Tirunelveli, India

**Keywords:** food, Ghana, modern, non-communicable diseases, outlets, retail, supermarket

## 1 Introduction

The study by Adjei et al. (1) provides timely and policy relevant evidence on the composition of modern retail food environments in the Greater Accra Region of Ghana. The authors surveyed a selection of supermarkets and mini marts across different districts, using shelf space measurements combined with the NOVA food classification system and energy density criteria to assess the availability of healthy and unhealthy foods. The survey reports that refined grain products (including instant noodles, biscuits, cakes, and cookies), sugar sweetened beverages, and alcoholic drinks occupied the largest proportions of available shelf space across the selected districts. In contrast, healthier food categories such as fresh fruits, fresh and unsalted vegetables, unprocessed staples, legumes, whole grains, fresh meat and fish products, and unsweetened beverages were minimally available and, in several outlets, entirely absent. Approximately 85 percent of total shelf space was allocated to unhealthy foods, while unprocessed or minimally processed foods constituted only a small fraction of in store availability. These empirically grounded findings underscore a structurally skewed retail food environment with important implications for dietary behavior and population health, particularly in rapidly urbanizing African contexts. This commentary reflects on the study's methodological contributions, public health relevance, and introduces the Retail Food Health Transition (RFHT) Framework as a conceptual extension to support future research and policy action.

## 2 Methodological rigor and analytical contributions

The study demonstrates methodological rigor through its objective quantification of retail shelf space and systematic categorization of food products using the globally recognized NOVA classification, complemented by energy density thresholds. Its adaptation of the International Network for Food and Obesity and NCD Research Monitoring and Action Support (INFORMAS) framework represents a significant methodological contribution, enabling cross country comparability and supporting policy monitoring efforts (15). The inclusion of both supermarkets and mini marts strengthens ecological validity and reflects the hybrid nature of Ghana's retail food ecosystem, where formal and informal outlets coexist (2). By quantifying floor and shelf areas, the authors advance African food environment research beyond descriptive observation toward objective measurement (3). One potential refinement lies in expanding the operationalization of “healthiness” to incorporate price accessibility and promotional exposure, both of which mediate purchasing behavior and dietary choices (4, 5). Integrating these dimensions could support the development of a multidimensional retail health index, enhancing policy relevance.

## 3 Socioeconomic context and policy implications

The study's examination of district level poverty incidence provides an important socioeconomic lens, revealing that districts with lower poverty incidence host more modern retail outlets but not necessarily healthier food assortments. This aligns with evidence from Brazil and South Africa, where supermarket density does not equate to nutritional equity (6, 7). In Ghana, retail modernization appears to increase exposure to energy dense, ultra processed foods (UPFs) rather than improving access to nutritious options (8, 9). These findings carry strong policy relevance. Multisectoral interventions involving the Ministry of Food and Agriculture, the Food and Drugs Authority, and municipal governments are necessary to promote healthier retail environments (14). Fiscal incentives could encourage retailers to allocate minimum shelf space to minimally processed foods, while front of pack labeling and zoning regulations could limit exposure to unhealthy products in sensitive locations (10).

## 4 Integrating theoretical perspectives: food system transition

The findings resonate strongly with nutrition transition theory, which links shifts from traditional diets to highly processed consumption patterns with globalization and urbanization (11). Ghana's retail transformation illustrates this global phenomenon in an African context. By connecting retail food availability with systemic drivers of dietary change, the study illuminates how obesogenic environments emerge and evolve in rapidly urbanizing economies. Future investigations could benefit from adopting systems thinking and complex adaptive systems approaches to trace feedback loops between consumer preferences, marketing practices, and regulatory policies. Such frameworks would enable the identification of leverage points, such as fiscal policies or supply chain reforms that could disrupt the structural dominance of UPFs.

## 5 Novelty and conceptual advancement

Building on the study's insights, this commentary introduces the Retail Food Health Transition (RFHT) Framework as a conceptual extension. The RFHT Framework delineates three stages of retail food environment transformation:

(a) Emergence stage: traditional markets dominate, and UPFs are limited.
(b) Expansion stage: supermarkets and mini marts proliferate, driving rapid growth in UPF availability.
(c) Regulatory reorientation stage: policy and market interventions rebalance shelf allocations toward healthier options.

This framework links micro level indicators (e.g., shelf space ratios of healthy vs. unhealthy foods) to macro level governance strategies, extending nutrition transition theory by emphasizing the structural determinants of retail food environments. By framing shelf space ratios as measurable indicators and potential policy levers, the RFHT Framework provides a practical tool for comparative studies across urbanizing regions in Sub Saharan Africa and for monitoring the impact of policy interventions.

## 6 Discussion and future scope

The original study establishes a valuable empirical baseline for Ghana's modern retail food environment. Future research should:

(a) Implement longitudinal audits to monitor shifts in shelf space ratios and product reformulations (12).
(b) Integrate consumer purchasing and dietary intake data to link retail availability with actual consumption outcomes (13).
(c) Apply geospatial mapping to identify clusters of unhealthy outlets in low income zones.
(d) Examine the influence of digital marketing and e-commerce platforms on UPF exposure.
(e) Test behavioral interventions, such as shelf repositioning or health signage, to promote healthier choices within existing retail layouts.

From a policy perspective, institutionalizing Retail Food Health Audits within urban planning and NCD prevention frameworks would enable systematic oversight of retail environments. Collaborative efforts between academia, government, and the private sector could facilitate data driven policies to improve nutritional equity. Inter country comparative studies would further enhance understanding of how African nations navigate the tension between retail modernization and public health, contributing to global initiatives like the Food Systems Countdown Initiative.

## 7 Conclusion

The original research represents a landmark contribution to empirical studies on African food environments, providing critical evidence on the structural dominance of UPFs in modern retail systems. This commentary highlights the study's methodological precision, contextual importance, and policy relevance, while the proposed RFHT framework advances theoretical integration by bridging micro level retail assessments with systemic policy reform. By promoting a multidimensional understanding of retail transitions, this commentary contributes to global discourse on reshaping food environments toward greater nutritional equity and sustainability in low and middle income countries. These insights can guide policymakers and retailers in designing interventions that increase the availability of healthier foods in urban Ghana.
